# Another Step Toward Early Ischemia Detection?∗

**DOI:** 10.1016/j.jacadv.2023.100449

**Published:** 2023-07-27

**Authors:** Jacqueline E. Joza

**Affiliations:** Department of Medicine, Division of Cardiology, McGill University Health Center, Montreal, Canada

**Keywords:** ischemia detection, personal health device, vectorcardiography

**Figure:**
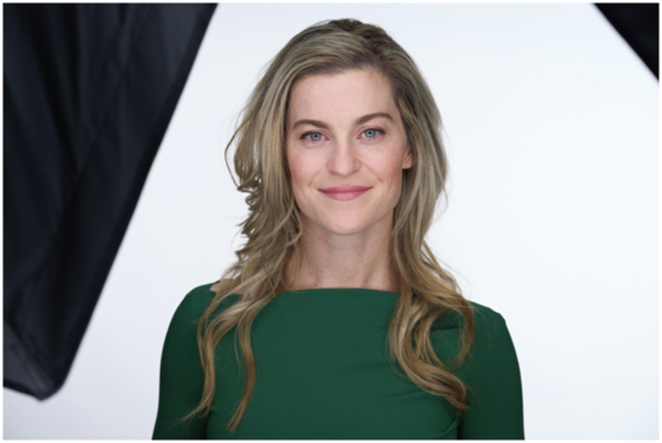

Approximately every 40 seconds, an American will have a myocardial infarction.^1^ A delay to coronary intervention with a door-to-balloon time of >90 minutes is associated with higher in-hospital, 30-day and 1-year mortality, and so the number of deaths that can be saved with early detection are countless.^2^ Personal health devices have gained immense traction over the last decade. An initial focus on arrhythmia conditions, wearables are now moving in the direction of early detection of coronary ischemia during acute myocardial infarction. With reliable self-detection of ongoing ischemia, earlier response times will improve outcomes.

In this issue of *JACC: Advances*, Shvilkin et al^3^ report the accuracy of a 3-lead “abc” electrocardiogram (ECG) system to detect ST-segment changes when compared to a standard 12-lead ECG. What is particularly clever is that this validation exercise was performed while actively ballooning the index coronary artery in a patient already undergoing planned percutaneous coronary intervention (PCI). The authors should be congratulated on this interesting and beautifully conducted study.

Consecutive patients undergoing PCI were fitted with a control 12-lead ECG system, as well as an additional 4 electrodes across the chest. Additional leads A and B were placed on the right and left shoulders respectively, with C and D matching below at chest level. This lead placement corresponds to the lead configuration that could eventually be implemented in a portable device with integrated electrodes, where right- and left-hand fingers produce a vector consistent with lead I (right-to-left ‘a’ lead), lead aVF (vertical ‘b’ lead), and a sagittal ‘c’ lead, creating the so-termed ‘abc’ lead ECG.

Patients then underwent 90-second balloon occlusion of the artery planned for PCI. Ten-second recordings of an ‘abc’ lead and standard 12-lead were taken at baseline, pre-inflation while the patient was on the table, and after 90-seconds of artery occlusion. The 90-second balloon occlusion could be stopped prematurely at 60 seconds in the presence of severe chest pain, hemodynamic instability, or ventricular arrhythmias. If more than 1 artery was targeted for PCI, then a second balloon occlusion of the other artery was performed with pre and post ECG recordings. Analyses of the ECGs were then performed using a vectrocardiographic approach for the ‘abc’ lead analysis, automated maximal single-lead ST-segment deviation for the standard 12-lead ECG, and standard 12-lead ECG interpretation by a cardiologist. Standard ischemic criteria were used based on the Fourth Universal Definition of Myocardial Infarction definition.

The ‘abc’ lead analysis used the initial portion of the ST-segment from the 10-second recordings. Using a vectorcardiographic approach by treating leads ‘a,’ ‘b,’ and ‘c’ as orthogonal, median QRST complexes and QRST vector loops were constructed. Two measurements were made: a ‘spot’ measurement which was calculated as the 3-dimensional abc-derived ST-segment vector loop segment deviation from an isoelectric point, and a ‘comparative’ measurement which measured the ST-segment vector loop segment difference between pairs of recordings (baseline, preocclusion and postocclusion). A heart rate correction was applied for the duration of the ST-segment duration. The 12-lead ECGs were digitally analyzed for average ST-segment deviation corrected for segment duration and heart rate. Similarly, both ‘spot’ and ‘comparative’ measurements were made with maximal ST-segment deviation with and without baseline subtraction respectively. Finally, 12-lead interpretation was performed by 3 board-certified cardiologists (1 interventionalist and 2 electrophysiologists), where they were tasked with evaluating if acute ischemia was present, Yes or No, in 3 scenarios: blinded ‘spot’ ECG reading, repeat ‘spot’ interpretation at 2 to 3 months later to assess for intra-observer variability, and an interpretation where the baseline ECG was clearly labeled, and compared to preinflation or inflation ECG: the so-called ‘comparative’ evaluation. The performance of the automated measurements was evaluated using receiver-operating characteristic curves.

A total of 66 patients were enrolled in the study, with only 23% having a normal baseline ECG and 42% with a baseline ST-segment deviation of >0.5 mm in at least 1 lead. A total of 120 balloon occlusions were performed where 50% underwent PCI to more than 1 artery. Interestingly, only 50 of 120 balloon occlusions performed resulted in an ST-segment change consistent with a definition for ischemia, and of those, 39 resulted in a ST-segment elevation consistent with a ST-segment elevation myocardial infarction definition. Left anterior descending arteryinflations produced larger ST-segment changes, although there were no significant differences in ST-segment deviation between inflations in the proximal, mid, and distal segments in any of the arteries.

There were several important findings in this study: 1) the comparative 3-lead “abc” system performed similarly to the comparative 12-lead ECG in detecting coronary artery occlusion, achieving over 90% sensitivity, specificity, and accuracy in determining coronary occlusion; 2) spot measurements demonstrated much poorer performance compared to comparative ones where a left anterior descending artery occlusion conferred a better performance as compared to the circumflex artery, although did not reach statistical significance. Importantly, however, none of the spot measurements missed any tracings matching the standard ischemia definition; and 3) only slightly more than half of the tracings were correctly classified by all 3 cardiologists, which increased to 68% when using the baseline comparison ECG. Further, more than 10% of tracings were misclassified by all 3 readers even despite the presence of a baseline for comparison.

There are important limitations to the study. First, it remains unclear as to why no ischemic changes were present in more than half of balloon occlusions: are we are missing a substantial amount of active ischemia based on ECG alone? Second, if this type of portable ECG analysis is to be integrated into daily life then validation needs to be performed in different patient populations with differing baseline characteristics, and tested in different patient positions, and at different heart rates simulating day-to-day life. Finally, baseline left bundle branch blocks were excluded however it was unclear how many patients had baseline conduction system disease which represents an important patient population to account for. Certainly the improvement in accuracy with a comparative vs spot ‘abc’-lead ECG suggests that when integrated into a smart-device, a daily baseline ‘abc’ lead ECG would likely have to be collected in various positions.

This is not the first portable 3-lead system to be introduced. Van Heuverswyn et al^4^ demonstrated the feasibility of their 3-lead detection (“RELF”) automatic algorithm built into a mobile handheld device for detection of acute coronary artery occlusion in patients who had been discharged with planned elective PCI. Self-recording was feasible for most patients with a sensitivity of 0.95 and 0.87 for an acute coronary artery occlusion with and without ECG changes respectively. Another smartphone ECG algorithm derives a 12-lead ECG equivalent from multiple single-lead recordings from a modified version of the AliveCor Heart Monitor paired with a fifth generation iPod Touch device.^5^ This has shown good correlation with the standard 12-lead ECG at time of ST-segment elevation myocardial infarction presentation.^5^ The Apple Watch is also capable of creating an ECG by positioning the smartwatch at various positions along the precordium creating 6 single-lead ECGs for V_1_ to V_6_ and has showed a strong correlation with the standard ECG.

The decision to proceed to coronary angiography is made on the complete patient presentation (history and physical exam, ECG, and focused bloodwork). However, we are fast entering into a new world where the ECG may soon take precedence. One can imagine in the not-so-distant future that smart-devices will be able to automatically detect active ischemia in the field, activate a rapid-response team that includes an ambulance and a cath lab team to provide streamlined access, skipping the emergency department altogether. Improving early detection and rapid response to a myocardial infarction is necessary; however, we need to proceed cautiously ensuring that the interpretation accounts for the patient as a whole.

## Funding Support and Author Disclosures

Dr Joza has received investigator-initiated external grant support from Medtronic Inc; consulting fees from Boston Scientific (modest); and honoraria from Biosense Webster Canada (modest).
